# Genetic diversity and population structure of *Vernonia amygdalina* Del. in Uganda based on genome wide markers

**DOI:** 10.1371/journal.pone.0283563

**Published:** 2023-07-26

**Authors:** Judith S. Nantongo, Juventine B. Odoi, Hillary Agaba, Samson Gwali

**Affiliations:** National Forestry Resources Research Institute, Kifu, Mukono, Uganda; National Cheng Kung University, TAIWAN

## Abstract

Determining the extent and distribution of genetic diversity is an essential component of plant breeding. In the present study, we explored the genetic diversity and population structure of *Vernonia amygdalina*, a fodder, vegetable and medicinal species of Africa and some parts of Yemen. Most empirical studies demonstrate that populations that are separated by geographic or ecological factors may experience genetic differentiation resulting from restricted gene flow between populations. A total of 238 individuals were sampled from two populations: i) Lake Victoria crescent (LVC) and ii) Southern and Eastern Lake Kyoga basin (SEK) agroecological zones of Uganda and genotyped using DArT platform. Of the two populations, the overall mean observed heterozygosity (Ho) was low to medium (Ho = 0.07[silicoDArTs] and 0.2[SNPs]). Inbreeding levels were also very low (-0.04 to -0.08) suggesting the presence of random mating. Partitioning of genetic structure in the two populations indicated that SEK exhibited a higher genetic diversity than LVC. The principal coordinates analysis (PCA) showed no geographical structuring, consistent with the low genetic differentiation (F_st_ = 0.00) and the low Euclidean genetic distance (1.38–1.39) between the LVC and SEK populations. However, STRUCTURE analysis with admixture models revealed weak possible genetic clusters with very small genetic distance among them. Overall, the results suggest low genetic diversity and weak genetic differentiation between the two populations. One possible explanation of the results could be the presence of human assisted gene flow over long distances.

## Introduction

Population genetic theory predicts that geographic distance will cause genetic differentiation among populations on the landscape, implying that populations that are near each other will often be more genetically similar, while distant populations are often more divergent [1, 2]. Geographic distances can limit allele exchange, for example through constraining seed and pollen dispersal in plants, producing geographically structured genetic variation [3, 4]. Various studies have showed the existence of correlations between genetic differentiation and geographic distance in many types of organisms [5, 6]. Genetic differentiation could be driven by natural forces even though current anthropogenic activities have caused a breakdown in structural connectivity (area and spatial configuration of habitats). By studying DNA sequence data from natural populations, research has begun to elucidate how these natural and anthropogenic processes are impacting on functional connectivity (gene flow) and genetic diversity of populations and hence their long-term survival.

Molecular markers have increasingly become popular as neutral tools for measuring genetic diversity and population structure [7, 8]. Genome reducing techniques such as Diversity Array Technology (DArT) (http://www.diversityarrays.com/) have improved the rate of genotype calling and the ability to sequence more samples for less cost [9]. Genotyping-by-sequencing (GBS) platform DArTSeq utilises Next-Generation-Sequencing to unravel the most informative representations of genomic DNA and thereby simplify marker discovery. DArTseq produces dominant (SilicoDArT) and co-dominant (SNP) markers that have been successfully applied for genetic structure analysis in several crops [10–12]. The markers allow the characterisation of population structure without prior knowledge of the genome or diversity [12–14]. Single nucleotide polymorphisms (SNPs) and SilicoDArT markers have become more popular for genetic analysis since they are ubiquitous in eukaryotic genomes and are bi-allelic in nature [15–18]. Therefore, using such molecular markers to characterise the genetic diversity of plant populations of interest can provide an efficient guidance for conservation.

*Vernonia amygdalina* Del (Asteraceae) (2n = 40) is a small perennial shrub growing predominantly in tropical African countries and some parts of Yemen on the Arabian Peninsula. It has dark green leaves and a rough bark, with height of up to 10 m. Its flowers are bisexual, regular, numerous, and strongly exserted (https://uses.plantnet-project.org/en/*Vernonia* *amygdalina* (PROTA)). The flower of *Vernonia* is considered to exhibit protandry, which imposes allogamy on this plant [19]. *Vernonia amygdalina* leaves, bark and roots are harvested for treatment of amoebic dysentery, gastrointestinal disorders, antimicrobial and antiparasitic activities [20, 21]. It is cultivated in some West African countries, where it is also consumed as a green leafy vegetable [20]. In Uganda, *Vernonia amygdalina* is especially important in the treatment of malaria [21], and accordingly several studies have documented the antimalarial properties of the different plant parts [20, 22]. *Vernonia amygdalina* has also been used to treat syphilis, ulcers, liver problems, tuberculosis, cough, abdominal pain, wounds, hernia and headache [21], hence it is important for bioprospecting.

In Uganda, *Vernonia amygdalina* occurs naturally in forest margins, woodlands and grasslands. It often occurs in disturbed localities such as abandoned farmland and can be found growing spontaneously in secondary forests [23]. Although no harvesting rates for this species have been estimated, various studies have shown that *V*. *amygdalina* is the most preferred species for malaria treatment in most parts of Uganda, including the study areas [21, 24]. This indirectly has an implication on the demand and, hence harvesting. In addition to its medicinal uses, it is also an important fodder species for domestic and wild animals in Uganda [25]. However, given the threat to natural areas from anthropogenic and non-anthropogenic causes, ex-situ conservation of this species is required. Since degradation of habitats has potential threats to the genetic integrity of most species, establishing the genetic structure of *V*. *amygdalina* can help to establish appropriate conservation, management, and sustainable utilization strategies [4, 26–28].

In spite of the high interest for *Vernonia amygdalina* among rural communities due to its therapeutical action on both bacterial and protozoal parasites [29], little is known on its population structure and genetic variation in Uganda. In the present study, genotyping-by-sequencing (GBS) using the DArTseq platform was used to genotype two geographically separated populations of *Vernonia amygdalina* in Uganda. The objectives were: 1) to assess genetic diversity in the two geographically isolated populations using SilicoDArT and SNP markers; 2) to investigate spatial genetic structure of *V*. *amygdalina*. We hypothesize that the geographical and ecological separation of *V*. *amygdalina* may cause genetic differentiation due to restricted gene flow between populations.

## Materials and methods

### Plant material

**Fig 1:**
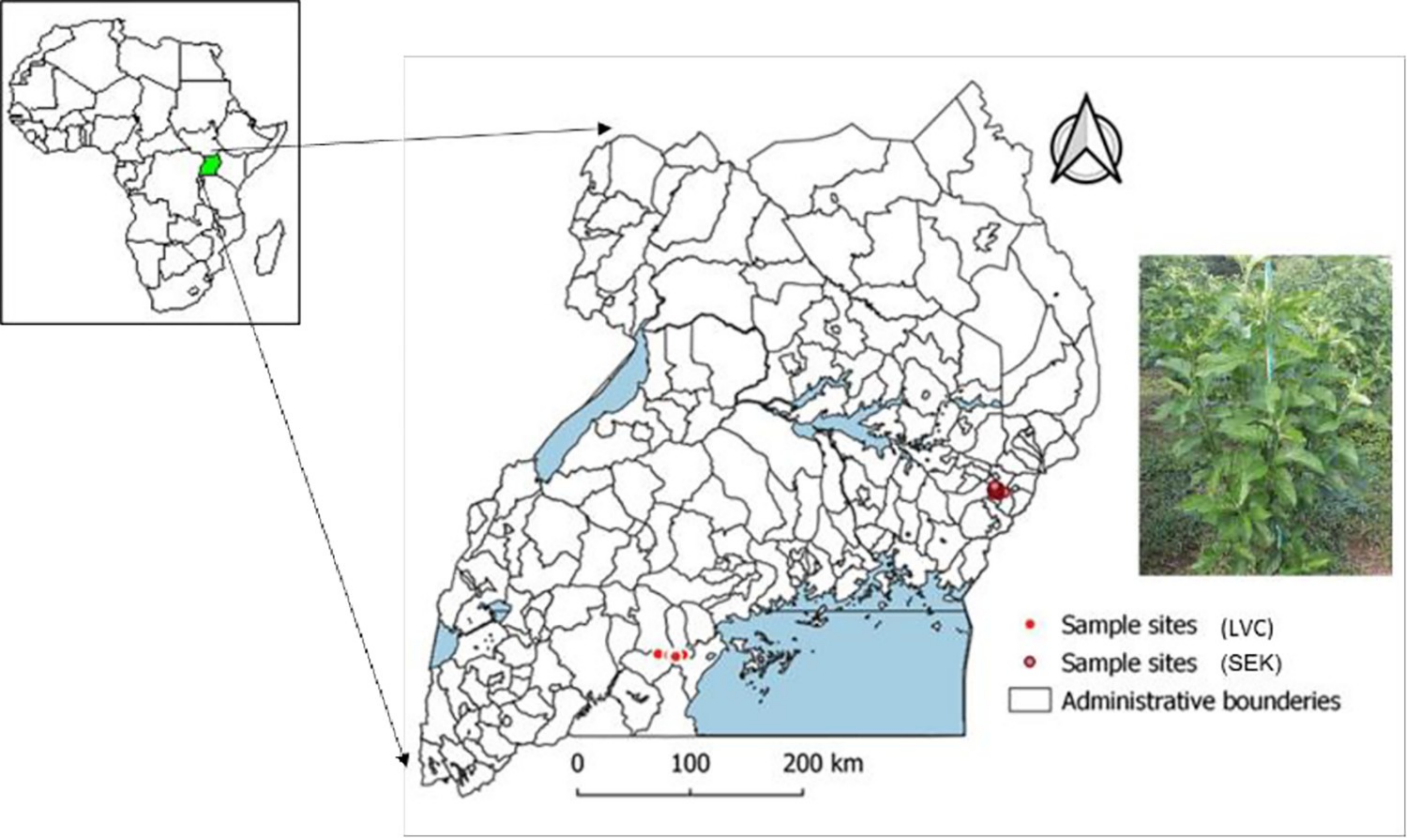
Map of Africa showing the location of Uganda and the map of Uganda showing the sites within the Lake Victoria crescent (LVC) and Southern and Eastern Lake Kyoga basin (SEK) agroecological zones where *V*. *amygdalina* (inset) samples were collected. The maps were generated in R.

Leaf samples were collected from trees in two agroecological zones; the Lake Victoria crescent (LVC, n = 120) and Southern and Eastern Lake Kyoga basin (SEK, n = 122), with specific coordinates, where samples were collected in S1 Table. The two populations will henceforth be referred to as LVC and SEK respectively. The geographical distance between the two sampling sites is approximately 353 km (Fig 1). Up to three young leaves were harvested from selected plants using a pair of scissors, which were cleaned with ethanol between each successive sampling. The sampled plants were at least 100m apart. The leaf samples were also cleaned using ethanol and immediately placed in a Ziplock bag with silica gel. The bags were stored in a cool box. The samples in silica gel were sent to Biosciences Eastern and Central Africa (BecA-ILRI) hub in Nairobi for DNA extraction.

### DNA extraction and DArTseq genotyping

DNA extraction was done on individual samples using Nucleomag plant genomic DNA extraction kit (Macherey-Nagel). The genomic DNA extracted was in the range of 50–100 ng/ul. DNA quality and quantity were checked on 0.8% agarose gel.

DNA was sent to Diversity Arrays Technology Pty Ltd laboratories in Canberra, Australia for sequencing using the HiSeq 2500 following the protocol optimised for *V*. *amygdalina*. DNA samples were processed individually in digestion/ligation reactions using a combination of PstI and HpaII Restriction Enzymes (RE) as described ealier [9, 12]. Briefly, mixed fragments” (PstI-HpaII) were amplified in 30 rounds of polymerase chain reaction (PCR) using the following reaction conditions: 94°C for 1 min, 30 cycles of; 94°C for 20 sec, 58°C for 30 sec, 72°C for 45 sec, followed by a final hold of 72°C for 7 min.

After PCR, equimolar amounts of amplification products from each sample of the 96-well microtiter plate were bulked and applied to c-Bot (Illumina) bridge PCR followed by sequencing on Illumina Hiseq2500. Sequences generated from each lane were processed using proprietary DArT analytical pipelines. SNP markers were aligned to the reference genomes of Chickpea_ICC_v2, Grape_v8 in the National Centre for Biotechnology Information (NCBI) in order to identify chromosome positions. The BLASTN algorithm with an e-value ≤ 5e-7 and an identity percentage of > = 90% was used. SilicoDArTs were scored as "dominant" markers, with "1" = Presence and "0" = Absence of a restriction fragment with the marker sequence in genomic representation of the sample. SNPs were scored as codominant markers with 0 for the homozygous allele aa, 1 for the heterozygous allele Aa and 2 for the homozygous allele AA. Finally, identical sequences were collapsed into “fastqcoll files”. The markers were tested for reproducibility (%)–the proportion of technical replicate assay pairs for which the marker score exhibited consistency; call rate (%)–the success of reading the marker sequence across the sample; polymorphism information content (PIC)—the degree of diversity of the marker in the population and the usefulness of the marker for linkage analysis; and one ratio–the proportion of the samples for which genotype scores equalled ‘1’.

### Genetic diversity analyses

The data were filtered using the dartR v 1.9.9.1 package [30] in R to remove all SNPs and silicoDArT markers that had > 5% missing data and individuals with > 10% missing data. Markers with a reproducibility score (RepAvg) < 100% were also removed as well as those that originated from the same fragment. Non-informative monomorphic markers including those with missing data were also removed. SNPs with a minor allele frequency (MAF) of < 1% were also discarded. MAF filtration was not done for presence/absence silicoDArT. Markers that could be adaptive were not excluded from the analysis [31]. The raw SNP data were deposited in figshare (10.6084/m9.figshare.21829035).

Analyses were done on the silicoDArT and SNP markers that were retained after the filtration above using the two populations (LVC and SEK). All genetic diversity indices were estimated using the R package “ADEGENET” [32]. The R package ADEGENET uses discriminant analysis of principal components to allow for data dimensionality reduction in large genomic datasets. The following diversity indices were therefore computed to illustrate the overall genetic divergence among the subpopulations: observed (H_o_) and expected heterozygosity (H_e_), total gene diversity (H_t_), genetic differentiation (F_st_) and population inbreeding coefficient (F_is_). Marker allele frequency–the frequency at which the second most common allele occurs in a given population [33], was also computed as the number of minor alleles in the population/total number of alleles in the population. The allelic richness (number of alleles in a population) and Shannon information index were also estimated using dartR package. Genetic distances were based on the Euclidean distance measure.

### Population structure analyses

To explore the geographical structuring of the genetic variation among individuals, a supervised principal coordinate analyses (PCoA) using dartR was used. PCoA was performed separately on the SNP and SilicoDArT datasets. To further examine the genetic structure of the populations that could be independent of geographical location, an unsupervised model-based Bayesian clustering was conducted using STRUCTURE 2.3.4 software using only SNP markers. The STRUCTURE program uses a Markov Chain Monte Carlo (MCMC) algorithm to cluster individuals into genetic populations on the basis of multilocus genetic data [34]. The analysis was run separately for silicoDArT and SNPs. Numbers in the range from 1 to 10 were assumed for K, since the micro-genetic structure of the species is unknown. The initial burn-in period, for each run, was set to 100,000 with 100,000 Markov chain Monte Carlo (MCMC) iterations [35]. This was replicated 5 times. The admixture model was applied without using any prior population information. To find the optimal value of K, the number of clusters (K) was tested in the range from 1 to 10, and were then plotted against ΔK in STRUCTURE HARVESTER [36] to identify the most likely value of K. The 10 runs of the optimal value of K were summarized using CLUMPP [37]. Further genetic analyses were explored based on clusters instead of geographically defined populations. Analysis was done up to the K value when all individuals were confidently placed in a cluster.

## Results

### SilicoDArT and SNP detection in *V*. *amygdalina*

A total of 8088 and 5429 silicoDArT and SNPs markers respectively were generated from 234 individuals of *V*. *amygdalina*. The call rate of the silicoDArT markers (average = 0.97) was higher than that of SNP markers (average = 0.78). Similarly, the reproducibility of the silicoDArT markers was higher (averaged to 1.0) than that of the SNP markers (average = 0.98). However, the average one ratio estimated for silicoDArTs (0.22) was similar to the SNPs (0.21) (Table 1).

**Table 1:** Minimum, maximum and average of marker quality parameters assessed for silicoDArTs and SNPs.

	silicoDArTs			SNPs		
	Min	Max	Average	Min	Max	Average
**Call rate**	0.80	1.00	0.97	0.36	1.00	0.78
**One ratio**	0.00	1.00	0.22	0.00	1.00	0.37
**Reproducibility**	0.95	1.00	1.00	0.90	1.00	0.98

### Genetic diversity

**Fig 2:**
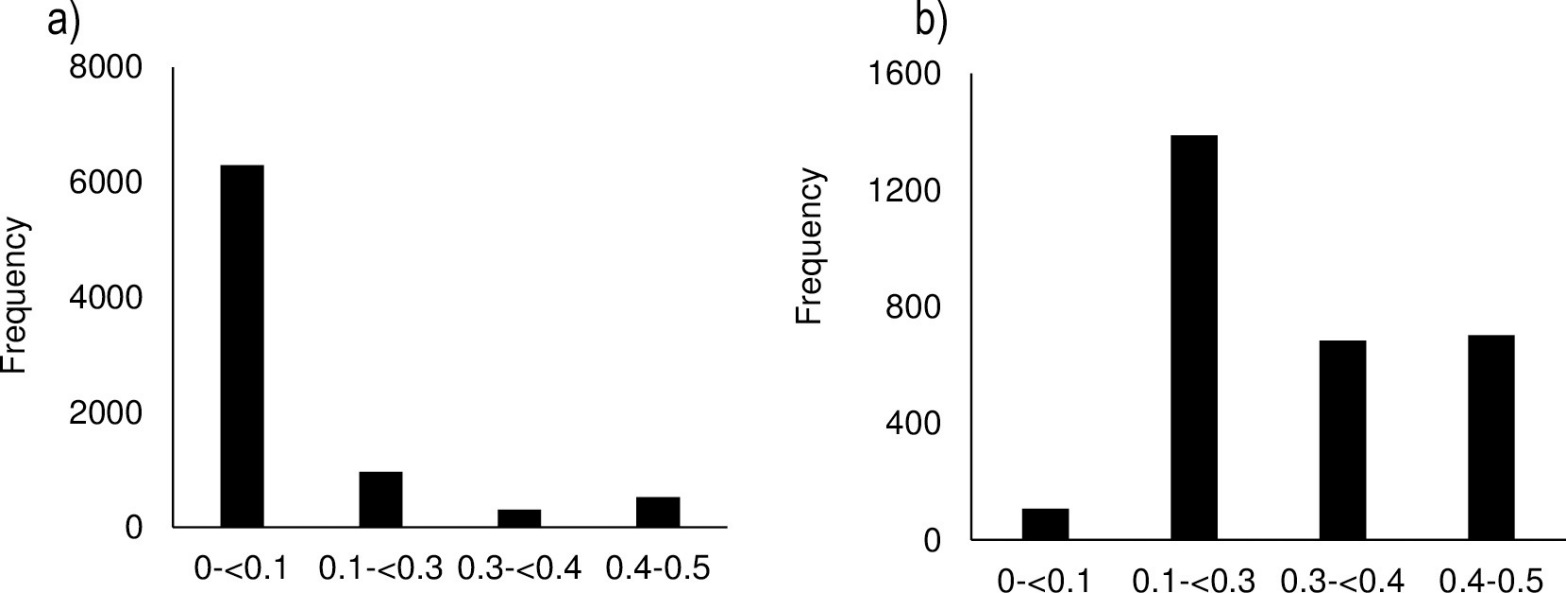
The polymorphic information content of the a) silicoDArT and b) SNP markers before data filtration.

The PIC value for SilicoDArT markers ranged from 0.02–0.5 (average = 0.08) and was lower than that of SNPs (range = 0–0.50, average = 0.21) for unfiltered data. The proportion of informative markers was higher for SNPs than silicoDArTs (Fig 2).

After filtering the data, all individuals and 5084 (62%) of silicoDArT markers were retained. For SNPs, 234 individuals and 1722 (31.7%) markers were retained. These were used for the proceeding analyses. The mean minor allele frequency (MAF) based on SNPs ranged between 0.002–0.5 with an average of 0.13. Only 43% of the SNP markers had minor allele frequency less than 0.05. MAF was not estimated for the dominant silicoDArT markers. After filtration, the PIC estimates ranged between 0–0.5 (average = 0.04) for silicoDArTs and 0–0.5 (average = 0.23) for SNPs.

Based on the two populations (LVC and SEK), the estimates for genetic diversity and differentiation were generally low for both markers. The genetic diversity calculated as expected heterozygosity (He) in the populations was 0.02 for silicoDArTs and 0.20 for SNPs (Table 2). Between the two populations, the diversity calculated as allelic richness, Shannon information index and heterozygosity was also low (Table 3).

**Table 2:** Genetic diversity of *V*. *amygdalina* based on silicoDArT and SNP markers. The populations were defined by the geographical origin (Lake Victoria cresecent-LVC and Southern and Eastern Lake Kyoga basin-SEK). Estimates with p indicate that these are corrected e.g. corrected Fst = Fstp.

	silicoDArTs	SNPs
**H**_**o**_	0.02	0.20
**H**_**e**_	0.02	0.19
**H**_**t**_	0.02	0.19
**H**_**tp**_	0.02	0.19
**D**_**st**_	0.00	0.00
**D**_**stp**_	0.00	0.00
**F**_**st**_	0.00	0.00
**F**_**stp**_	0.00	0.00
**F**_**is**_	-0.08	-0.04
**D**_**est**_	0.00	0.00

**Table 3:** Allelic richness ± standard deviation, Shannon information index ± standard deviation and heterozygosity ± standard deviation of the two geographically defined populations estimated from SNP markers. The populations were defined by the geographical origin (Lake Victoria crescent-LVC and Southern and Eastern Lake Kyoga basin-SEK). These parameters cannot be generated from dominant silicoDArT markers.

	nloci	Allelic richness	Shannon information index	Heterozygosity
**LVC**	1722	0.80 ± 0.40	0.29 ± 0.25	0.18 ± 0.18
**SEK**	1722	0.88 ± 0.32	0.30 ± 0.24	0.18 ± 0.17

### Geographical genetic differentiation

**Fig 3:**
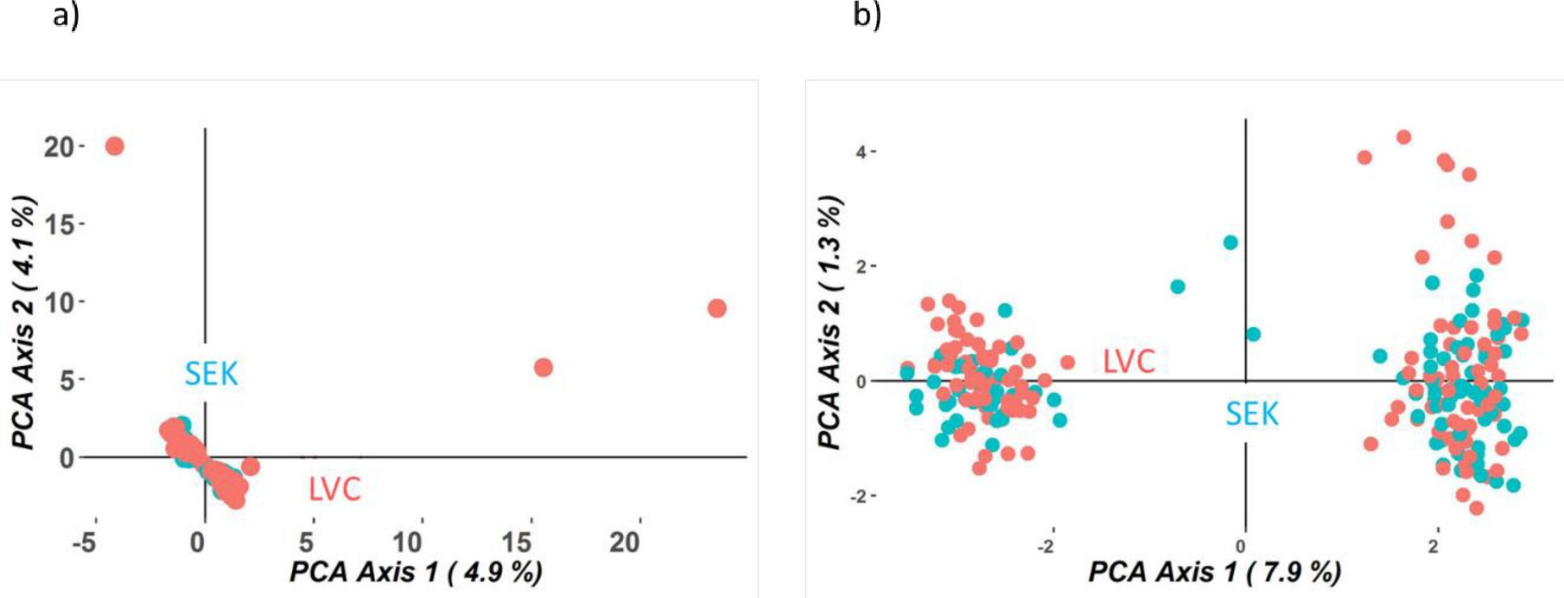
Principal coordinates analysis plot to infer group structure of *V*. *amygdalina* based on a) silicoDArT b) SNP markers. The populations were defined by the geographical origin (Lake Victoria crescent-LVC and Southern and Eastern Lake Kyoga basin-SEK).

Genetic relationships among the individuals from LVC and SEK explored by principal coordinates analysis (PCoA) (Fig 3) indicated that the two populations could not be clearly separated. Irrespective of location, there are two possible groups (clusters) that occur in both regions. The principal coordinates explained little variation between the populations, where the first principal coordinate axis explained only 4.9% [silicoDArTs] and 7.9% [SNPs]. The second principal coordinate axis explained 4.1% [silicoDArTs] and 1.3% [SNPs]. The Euclidean genetic distance estimated based on geographical populations was also low for both silicoDArTs (1.38) and SNPs (1.39). Consistently, an Fst value of 0.00 was estimated, suggesting no genetic differentiation between these two populations (Table 2). Very low coefficients of inbreeding (-0.08 [silicoDArTs], -0.04[SNPs]) were also estimated.

Based on unsupervised clustering of SNP markers in STRUCTURE, the optimal K-value showed the possibility of having two subpopulations, consisting of 45% and 55% of individuals (S1 Fig). However, in addition, there was a small peak observed at K = 4 (S1 Fig), which might indicate another informative population structure. Therefore, the STRUCTURE results at K = 2, 3 and K = 4 were subject to additional genetics analyses. Still, low genetic diversity estimates were generated, when K was assumed to be 2, 3 or 4 (S2 Table). There was no clear separation detected by the PCOa using these clusters (S2 Fig data not shown), suggesting a lack of genetic structuring among between the two sampled populations, and that the clusters are possibly artifacts. However, the genetic estimates improved by using the clusters instead of the geographically defined populations (Table 4, S3 Table). The Euclidean genetic distances also increased with the number of clusters, where distance was 4.5 (K = 2), 6.9 (K = 3) and 7.0 (K = 4) (S4 Table). The results also showed an admixture of individuals between/among the clusters, where some individuals were placed in two or more groups (S2–S4 Figs). Genetic indices within and between individual clusters also indicate high levels of genetic differentiation among individuals of some clusters (S4 Table) but not among the clusters. However, very small genetic distances were detected among individuals of the same clusters (S5 Table). Only SNP markers were used for STRUCTURE analysis.

**Table 4:** Allelic richness ± standard deviation, Shannon information index ± standard deviation and heterozygosity ± standard deviation of the STRUCTURE defined clusters (K = 2) estimated from SNP markers. Number of loci (nloci) = 1722. Individuals that were not significantly placed in either cluster were discarded during these analyses.

	1	2
proportion membership (%)	55	45
expected heterozygosity	0.18	0.19
Genetic differentiation	0.16	0.10
Allelic richness	0.93 ± 0.26	0.92 ± 0.28
Shannon information index	0.37 ± 0.24	0.38 ± 0.25
Heterozygosity	0.25 ± 0.18	0.25 ± 0.19

## Discussion

Based on silicoDArT and SNP markers, the results indicated; (i) low to medium genetic variation in *V*. *amygdalina* with potential consequences on the species ability to recover from demographic, environmental and genetic stochasticity [28]. (ii) no genetic differentiation between the two populations samples in the two geographical areas. Irrespective of location, two possible genetic groups exist.

### Low genetic diversity

The low genetic diversity estimates observed with silicoDArTs could have resulted from the very low PIC values (0.08) exhibited by these markers. PIC is often underestimated for dominant markers. Informativeness of markers based on PIC can be low (0 to 0.10), medium (0.10 to 0.25), high (0.30 to 0.40) and very high (0.40 to 0.50) [38, 39]. In contrast SNPs exhibited medium to informativeness (average PIC = 0.21) suggesting that they can detect the polymorphism among the individuals of *V*. *amydalina*. The silicoDArTs PIC value was lower than what has been established for tropical plants [12]. For SNPs, the PIC value was in the range for other trees in the same region like *Trema orientalis* (average PIC = 0.27) [12], suggesting that SNPs are a better predictor of genetic diversity than silicoDArTs. In theory, larger populations of *V*. *amygdalina* should hold more genetic variation than the small populations (Amos and Harwood 1998) such as *T*. *orientalis*, which was not the case [40]. The low PIC values corroborate with the low measures of allelic richness and Shannon information index, signifying low genetic diversity for populations. The low PIC values in this study contrasted high estimates in other *V*. *amygdalina* populations [41] indicating presence of population specific bottlenecks.

The low to average genetic variation in *V*. *amygdalina* could be due to reduction in the occurrence of sexual reproduction, resulting from the frequent removal of young twigs for medicinal use and forage by domestic and wild animals. Removing young twigs reduces flower production that consequently reduces the effective population available for crossing. Under a neutral model, effective population size is a key factor that determines population’s genetic diversity [42]. Additionally, in areas where *V*. *amygdalina* is frequently harvested in the wild, the plants tend to reproduce through root vegetative reproduction. Asexual reproduction is predicted to experience reduced genetic variation as a result of the absence of segregation and genetic recombination [43].

The very low inbreeding coefficient estimated based on geographical location is consistent with the possible lack of flowering mentioned above. The reduced genetic variation could also be explained by the premise that *V*. *amygdalina* is expanding its range as a result of enlargement of disturbed localities and secondary forests, where the plant prefers to grow, or introductions by humans into previously unoccupied areas as they transport medicine from one place to another. Populations that are the result of range expansions into previously unoccupied areas may have lower levels of genetic diversity as a result of repeated founder events [44]. This is also suggested by the slight negative Fis. The low genetic diversity within a population is widely acknowledged to lead to a reduction in adaptive potential, which may increase extinction risk [28].

### Geographical structuring of genetic variation

In this study, the supervised PCOa did not clearly separate the two populations. This was consistent with the very low genetic differentiation coefficient (F_st_; SNPs = 0.0008, silicoDArTs = 0.0005) suggesting that the 353 km distance and other geographical barriers such as river Nile between the study sites is not a constraint to gene flow in this species. Ideally, F_st_ values below 0.05 indicate low genetic differentiation, while values between 0.05–0.15, 0.15–0.25, and above 0.25 indicate moderate, high, and very high genetic differentiation respectively [45]. The near-zero F_is_ estimate based on geographically determined populations signifies the presence of random mating also known as Hardy–Weinberg equilibrium, and that the available parents experience a high level of outcrossing leading to homogenization of the populations. Concerning the mode of fertilization, the flower of Vernonia is considered to exhibits protandry, which imposes allogamy on this plant [19].This also implies that the mode of dispersal between the two populations is quite efficient.

The spatial scales for causing genetic differentiation in plant species vary greatly, dependent on factors such as biology of the species, geography among others. Durka, Michalski [46] for example indicated that genetic differentiation may occur at scales of 3.5–800 km. The literature discussing pollination modes of *V*. *amygdalina* gives the impression that the species is pollinated by insects (Dumas et al. 2017). Although pollen flow is commonly skewed toward short distances of just a few meters, reflecting insect behaviour and the spacing of the plants, there is increasing evidence that small flying insects can disperse over large distances [47]. Given that *V*. *amygdalina* occurs naturally in mostly open places; forest margins, woodlands and grasslands or disturbed localities such as abandoned farmland and secondary forests makes it prone to frequent visits by pollinators, and hence the weak spatial structure. There also seems to be a constraint on self-pollination in *V*. *amygdalina*, where the plant seems to be allogamous [19]. Self-incompatibility is one of the evolutionary adaptations that promotes high out-crossing rates in plant species [4]. In addition to natural factors, the absence of genetic differentiation strongly suggests the presence of human-assisted long-distance gene flows. Whether the migration was from Lake Victoria crescent-LVC to Southern and Eastern Lake Kyoga basin-SEK or vice versa may be an interesting subject of investigation. The plant is very commonly used in the treatment of various diseases especially malaria [20]. The widespread effects of malaria and other diseases in Uganda may have driven its dispersal by humans from a few natural locations to other areas. The results of this current study however, contrast observations in other *V*. *amygdalina* populations where, geographical distinctness with a possible effect of plant isolation by distance and restricted gene flow were observed among the accessions [41].

The unsupervised Bayesian clustering algorithm implemented in STRUCTURE clustered individuals into different numbers of clusters, possibly suggesting presence of short-scale genetic differentiation. It is also possible that the clustering results from sampling of different ‘varieties’. Elsewhere, some variations have been observed in *V*. *amygdalina*, based on the level of bitterness, which ranges from very bitter to less bitter, with the ‘bitter’ type possessing a deep green coloration and a deep bitter taste and the ‘less bitter’ type possessing a fairly light green coloration with little or no bitter taste [41]. Although, these differences are also locally known for Uganda *V*. *amygdalina*, they were not considered during the sampling for this study. Future studies possibly need to genetically characterise these ‘bitterness’ groups separately for further understanding of genetic relationships among *V*. *amygdalina*. There was, however, still low genetic differentiation between/among the different clusters, which allows us to assume that the two sampled populations constitute a single genetic population cohesive probably by the movement of pollen by insects and by the use that the local population gives to this species. The high positive F_IS_ values and small genetic distances suggest presence of high inbreeding between and among individuals of different clusters. One possible explanation of this observation could be that insect mediated mating occurs among specific relatives, possibly over short distances to cause inbreeding, but human assisted gene flow causes homogenisation of genetic structure over long distances. It is also possible that the individuals in both LVC and Southern and SEK are remnants of a single population of much greater density, where related individuals had greater opportunity to interbreed.

### Management implications

The low levels of genetic diversity in the studies *V*. *amygdalina* populations could decrease their ability to cope with anthropogenic and other environmental threats, leading to species endangerment or even extinction. Deliberate efforts to reduce overuse, as well as enhancing seed production for example through controlled crosses could improve genetic diversity. However, characterizing the genetic diversity of other populations, that could be more genetically diverse as well as considering potential phenotypic differences of ‘varieties’ will be important for making core collections for ex-situ management. It is acknowledged that where there is significant genetic differentiation among populations or groups of populations, they should be managed as distinct entities. In our case, Lake Victoria Crescent (LVC) and Southern and Eastern Lake Kyoga basin-SEK populations can be treated as the same population, for making core collections and for conservation. This study also suggests that geographical distance between populations should not necessarily be considered as the sole or best determinant of gene flow levels among populations of plants.

## Supporting information

S1 Fig Delta K (ΔK) for different numbers of subpopulations (K) based on SNP markers. (DOCX)

S2 Fig **a)**. Principal coordinates analysis plot to infer group structure of *V*. *amygdalina* based on SNP markers. The populations were defined by clusters identified in STRUCTURE, where K = 2. Pink = individuals placed in cluster 1, blue = individuals placed in cluster 2, grey = individuals not significantly placed in either cluster b) estimated population structure of V. amygdalina individuals on K = 2. Accessions in blue were clustered into cluster 1(red, n = 55%) and cluster 2(green, n = 45%). **b)** estimated population structure of V. amygdalina individuals on K = 2. Individuals were clustered into cluster 1(red) and cluster 2(green).
(DOCX)

S3 Fig **a)** Principal coordinates analysis plot to infer group structure of *V*. *amygdalina* based on SNP markers. The populations were defined by clusters identified in STRUCTURE, where K = 3. 1 = individuals placed in cluster 1, 2 = individuals placed in cluster 2, 3 = individuals placed in cluster 3, 4 = individuals placed in clusters 1 & 2, 5 = individuals placed in clusters 2 & 3, grey = individuals not significantly placed in either cluster. **b)** estimated population structure of *V*. *amygdalina* individuals on K = 3. Individuals were clustered into cluster 1(red, n = 55%), 2(blue, n = 44%) and 3 (green, n = 1%).
(DOCX)

S4 Fig **a)** Principal coordinates analysis plot to infer group structure of *V*. *amygdalina* based on SNP markers. The populations were defined by clusters identified in STRUCTURE, where K = 3. 1 = individuals placed in cluster 1, 2 = individuals placed in cluster 2, 3 = individuals placed in cluster 3, 4 = individuals placed in clusters 1 & 2, 5 = individuals placed in clusters 2 or 3 different clusters. **b)** estimated population structure of *V*. *amygdalina* individuals on K = 3. Individuals were clustered into cluster 1(green, n = 54%), 2(blue, n = 44%), 3 (red, n = 0.01%) and 4 (yellow, n = 0.01%).
(DOCX)

S1 Table GPS location (Northings and Eastings) of the leaf samples collected from Masaka (Lake Victoria Crescent (LVC) and Mbale (Southern and Eastern Lake Kyoga basin-SEK). (XLSX)

S2 Table Genetic diversity of *V*. *amygdalina* based on SNP markers. Populations in this case were defined by STRUCTURE. Individuals that were not significantly placed in either cluster were discarded during these analyses (See S1–S4 Figs).
(DOCX)

S3 Table Allelic richness ± standard deviation, Shannon information index ± standard deviation and heterozygosity ± standard deviation of the STRUCTURE defined clusters estimated from SNP markers. Individuals that were not well placed in the different clusters were excluded during the estimations. Number of loci = 1722. Individuals that were not significantly placed in either cluster were discarded during these analyses.
(DOCX)

S4 Table The number of individuals placed in each cluster including the expected heterozygosity and genetic differentiation between individuals. The results are based on SNP markers.
(DOCX)

S5 Table Genetic distances between clusters based on SNP markers. (DOCX)
