# Supplementary material for: Genetic diversity and population structure of Vernonia amygdalina Del. in Uganda based on genome wide markers
Source: PLoS One. 2023 Jul 26;18(7):e0283563. doi: 10.1371/journal.pone.0283563 (PMC10370736; doi:10.1371/journal.pone.0283563)
Supplement: S1 Fig — (DOCX) [file pone.0283563.s001.docx]

**Supplementary Figure S1:** Delta K (ΔK) for different numbers of subpopulations (K) based on SNP markers.
