# Supplementary material for: Genetic diversity and population structure of Vernonia amygdalina Del. in Uganda based on genome wide markers
Source: PLoS One. 2023 Jul 26;18(7):e0283563. doi: 10.1371/journal.pone.0283563 (PMC10370736; doi:10.1371/journal.pone.0283563)
Supplement: S2 Fig — a). Principal coordinates analysis plot to infer group structure of V. amygdalina based on SNP markers. The populations were defined by clusters identified in STRUCTURE, where K = 2. Pink = individuals placed in cluster 1, blue = individuals placed in cluster 2, grey = individuals not significantly placed in either cluster b) estimated population structure of V. amygdalina individuals on K = 2. Accessions in blue were clustered into cluster 1(red, n = 55%) and cluster 2(green, n = 45%). b) estimated population structure of V. amygdalina individuals on K = 2. Individuals were clustered into cluster 1(red) and cluster 2(green). (DOCX) [file pone.0283563.s002.docx]

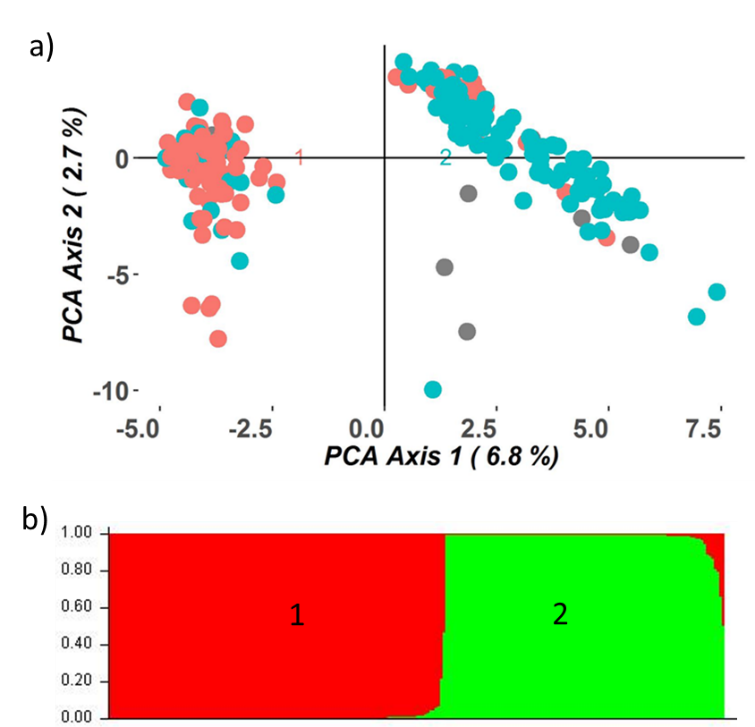


**Supplementary Figure S2: a).** Principal coordinates analysis plot to infer group structure of *V. amygdalina* based on SNP markers. The populations were defined by clusters identified in STRUCTURE, where K= 2. Pink= individuals placed in cluster 1, blue= individuals placed in cluster 2, grey= individuals not significantly placed in either cluster b) estimated population structure of V. amygdalina individuals on K = 2. Accessions in blue were clustered into cluster 1(red, n = 55%) and cluster 2(green, n = 45%). **b)** estimated population structure of V. amygdalina individuals on K = 2. Individuals were clustered into cluster 1(red) and cluster 2(green).
