# Supplementary material for: Genetic diversity and population structure of Vernonia amygdalina Del. in Uganda based on genome wide markers
Source: PLoS One. 2023 Jul 26;18(7):e0283563. doi: 10.1371/journal.pone.0283563 (PMC10370736; doi:10.1371/journal.pone.0283563)
Supplement: S4 Fig — a) Principal coordinates analysis plot to infer group structure of V. amygdalina based on SNP markers. The populations were defined by clusters identified in STRUCTURE, where K = 3. 1 = individuals placed in cluster 1, 2 = individuals placed in cluster 2, 3 = individuals placed in cluster 3, 4 = individuals placed in clusters 1 & 2, 5 = individuals placed in clusters 2 or 3 different clusters. b) estimated population structure of V. amygdalina individuals on K = 3. Individuals were clustered into cluster 1(green, n = 54%), 2(blue, n = 44%), 3 (red, n = 0.01%) and 4 (yellow, n = 0.01%). (DOCX) [file pone.0283563.s004.docx]

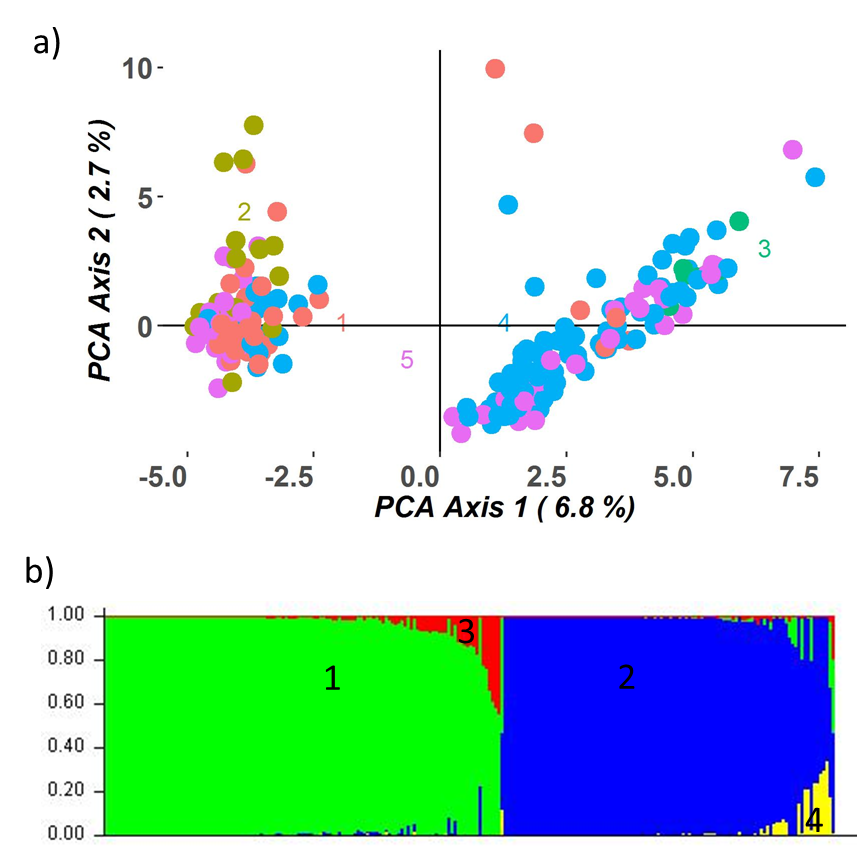


**Supplementary Figure S4: a)** Principal coordinates analysis plot to infer group structure of *V. amygdalina* based on SNP markers. The populations were defined by clusters identified in STRUCTURE, where K= 3. 1 = individuals placed in cluster 1, 2 = individuals placed in cluster 2, 3 = individuals placed in cluster 3, 4 = individuals placed in clusters 1 & 2, 5= individuals placed in clusters 2 or 3 different clusters. **b)** estimated population structure of *V. amygdalina* individuals on K = 3. Individuals were clustered into cluster 1(green, n = 54%), 2(blue, n = 44%), 3 (red, n = 0.01%) and 4 (yellow, n = 0.01%)
