# Supplementary material for: Genetic diversity and population structure of Vernonia amygdalina Del. in Uganda based on genome wide markers
Source: PLoS One. 2023 Jul 26;18(7):e0283563. doi: 10.1371/journal.pone.0283563 (PMC10370736; doi:10.1371/journal.pone.0283563)
Supplement: S2 Table — Populations in this case were defined by STRUCTURE. Individuals that were not significantly placed in either cluster were discarded during these analyses (See S1–S4 Figs). (DOCX) [file pone.0283563.s006.docx]

**Supplementary Table S2:** Genetic diversity of *V. amygdalina* based on SNP markers. Populations in this case were defined by STRUCTURE. Individuals that were not significantly placed in either cluster were discarded during these analyses (See supplementary figures).

|  | K=2 | K=3 | K=4 |
| --- | --- | --- | --- |
| H_o_ | 0.07 | 0.07 | 0.07 |
| H_e_ | 0.25 | 0.25 | 0.25 |
| H_t_ | 0.26 | 0.26 | 0.26 |
| H_tp_ | 0.26 | 0.27 | 0.27 |
| D_st_ | 0.00 | 0.02 | 0.01 |
| D_stp_ | 0.01 | 0.02 | 0.02 |
| F_st_ | 0.02 | 0.06 | 0.05 |
| F_stp_ | 0.04 | 0.09 | 0.07 |
| F_is_ | 0.71 | 0.72 | 0.73 |
| D_est_ | 0.01 | 0.03 | 0.03 |
