# Supplementary material for: Genetic diversity and population structure of Vernonia amygdalina Del. in Uganda based on genome wide markers
Source: PLoS One. 2023 Jul 26;18(7):e0283563. doi: 10.1371/journal.pone.0283563 (PMC10370736; doi:10.1371/journal.pone.0283563)
Supplement: S3 Table — Individuals that were not well placed in the different clusters were excluded during the estimations. Number of loci = 1722. Individuals that were not significantly placed in either cluster were discarded during these analyses. (DOCX) [file pone.0283563.s007.docx]

**Supplementary Table S3**: Allelic richness ± standard deviation, Shannon information index ± standard deviation and heterozygosity ± standard deviation of the STRUCTURE defined clusters estimated from SNP markers. Indidulas that were not well placed in the different clusters were excluded during the estimations. Number of loci =1722. Individuals that were not significantly placed in either cluster were discarded during these analyses.

|  |  | Allelic richness | Shannon information index | Heterozygosity |
| --- | --- | --- | --- | --- |
| K=2 | 1 | 0.93 ± 0.26 | 0.37 ± 0.24 | 0.25 ± 0.18 |
|  | 2 | 0.92 ± 0.28 | 0.38 ± 0.25 | 0.25 ± 0.19 |
| K=3 | 1 | 0.83 ± 0.37 | 0.38 ± 0.26 | 0.25 ± 0.19 |
|  | 2 | 0.92 ± 0.27 | 0.38 ± 0.25 | 0.25 ± 0.19 |
|  | 3 | 0.67 ± 0.47 | 0.33 ± 0.27 | 0.22 ± 0.19 |
| K=4 | 1 | 0.87 ± 0.34 | 0.39 ± 0.25 | 0.26 ± 0.19 |
|  | 2 | 0.74 ± 0.44 | 0.37 ± 0.27 | 0.25 ± 0.19 |
|  | 3 | 0.55 ± 0.50 | 0.30 ± 0.29 | 0.21 ± 0.20 |
|  | 4 | 0.88 ± 0.32 | 0.37 ± 0.26 | 0.24 ± 0.19 |
