# Supplementary material for: Genetic diversity and population structure of Vernonia amygdalina Del. in Uganda based on genome wide markers
Source: PLoS One. 2023 Jul 26;18(7):e0283563. doi: 10.1371/journal.pone.0283563 (PMC10370736; doi:10.1371/journal.pone.0283563)
Supplement: S4 Table — The results are based on SNP markers. (DOCX) [file pone.0283563.s008.docx]

**Supplementary Table S4**: The number of individuals placed in each cluster including the expected heterozygosity and genetic differentiation between individuals. The results are based on SNP markers

|  |  | proportion membership (%) | expected heterozygosity | Genetic differentiation |
| --- | --- | --- | --- | --- |
| K=1 |  | 100 | 0.19 | 0.30 |
| K=2 | 1 | 55 | 0.18 | 0.16 |
|  | 2 | 45 | 0.19 | 0.10 |
|  |  |  |  |  |
| K=3 | 1 | 55 | 0.18 | 0.17 |
|  | 2 | 44 | 0.19 | 0.11 |
|  | 3 | 1 | 0.24 | 0.30 |
|  |  |  |  |  |
| K=4 | 1 | 53 | 0.18 | 0.17 |
|  | 2 | 44 | 0.19 | 0.11 |
|  | 3 | 2 | 0.17 | 0.56 |
|  | 4 | 1 | 0.23 | 0.34 |
