# Supplementary material for: Genetic diversity and population structure of Vernonia amygdalina Del. in Uganda based on genome wide markers
Source: PLoS One. 2023 Jul 26;18(7):e0283563. doi: 10.1371/journal.pone.0283563 (PMC10370736; doi:10.1371/journal.pone.0283563)
Supplement: S5 Table — (DOCX) [file pone.0283563.s009.docx]

**Supplementary Table S5**. Genetic distances between clusters based on SNP markers

| **K=2** |  |  | **K=3** |  |  |  |  | **K=4** |  |  |  |  |
| --- | --- | --- | --- | --- | --- | --- | --- | --- | --- | --- | --- | --- |
|  | 1 |  |  | 1 | 2 | 3 |  |  | 1 | 2 | 3 | 4 |
| 1 | - |  | 1 | - | - | - |  | 1 | - | - | - | - |
| 2 | 0.01 |  | 2 | 0.01 | - | - |  | 2 | 0.02 | - | - | - |
|  |  |  | 3 | 0.02 | 0.03 | - |  | 3 | 0.05 | 0.05 | - | - |
|  |  |  |  |  |  |  |  | 4 | 0.03 | 0.03 | 0.06 | - |
